# Correlation Between Clinical and Biochemical Markers in Patients With Acromegaly on Different Modalities of Treatment

**DOI:** 10.7759/cureus.19438

**Published:** 2021-11-10

**Authors:** Qusay Baqer Alzajaji, Haider A Alidrisi, Abbas A Mansour

**Affiliations:** 1 Diabetes and Endocrinology, Faiha Specialized Diabetes, Endocrine and Metabolism Center (FDEMC), Basrah, IRQ; 2 Internal Medicine, University of Basrah, Basrah, IRQ; 3 Diabetes and Endocrinology, University of Basrah College of Medicine, Basrah, IRQ; 4 Diabetes and Endocrinology, Faiha Specialized Diabetes, Endocrine and Metabolism Center (FDEMC) University of Basrah, Basrah, IRQ

**Keywords:** quality of life, biochemical markers, clinical features, disease activity, acromegaly

## Abstract

Background

Acromegaly is a disabling disease caused by growth hormone (GH) over-secretion, associated with increased morbidity and mortality through different metabolic and somatic consequences involving soft tissue, acral overgrowth, and skin thickening, which will lead to different clinical manifestations. The study aimed to assess the correlation between biochemical markers of acromegaly with different clinical findings and biochemical control with the clinical findings.

Methods

A cross-sectional study was done on 56 patients with acromegaly attending a tertiary center for diabetes, endocrine, and metabolism in Southern Iraq Basrah. They were 32 (57.1%) males. Fatigue, headache, excessive sweating, joint pain, backache, soft tissue swelling, numbness, snoring, and visual problems were assessed on a four-point Likert scale. In addition, biochemical dynamic GH testing was done using a five-point random GH curve and/or standard GH suppression under the oral glucose tolerance test (OGTT).

Results

No significant correlation was found between symptoms severity and five-point GH parameters in respect of mean, peak, and nadir. Only backache showed a significant correlation with GH under OGTT suppression parameters in respect to mean, peak, and nadir (P-values < 0.01, < 0.01, and < 0.01), respectively. No particular biochemical control cut-off value in the 9 am random GH, mean five-point GH curve, or nadir GH under OGTT was correlated with the degree of severity for any of the clinical symptoms, as there was no difference between the biochemically controlled and uncontrolled groups in respect to any of the clinical symptom’s severity scale.

Conclusion

Only backache correlated with the biochemical tests of disease activity in the form of GH under OGTT. It appears in this study that the severity of the clinical symptoms does not correlate with biochemical control so that thorough evaluation and treatment of clinical complaints besides biochemical control is an essential part of the management plan in patients with acromegaly.

## Introduction

Acromegaly is a disabling disease caused by growth hormone (GH) over secretion and secondary insulin-like growth factor-I (IGF-I) excess [1]. The annual incidence of GH-secreting adenomas that cause acromegaly is three to four cases/million, with equal gender prevalence distribution and the mean age around the mid-forties [2]. In a local study, acromegaly constitutes 41% of the registered pituitary adenomas [3]. Due to the gradual disease onset and generally nonspecific symptoms, diagnosis is frequently delayed [2]. In addition, direct, somatic, and metabolic effects lead to different nonspecific signs and symptoms that may affect the quality of life, including headache, visual disturbance, soft tissue swelling, and acral enlargements with different consequent complications [2].

Pituitary adenomas may reduce the health-related quality of life (HrQoL) in patients with acromegaly. This reduction in HrQoL is inversely related to the duration of active acromegaly [4-6]. The SAGIT and ACRODAT instrument is a comprehensive outcome tool to evaluate acromegaly's fundamental features to assist in managing acromegaly in practice [7-8]. They combined symptomatology, comorbidities, biochemical indicators, and tumor profile [4]. The Acromegaly Quality of Life (AcroQoL) is a 22-item questionnaire divided into two scales; the first contains eight items for the physical aspect and the second includes 14 items for the psychological part of the HrQoL. This second scale is subdivided into two subscales, one for appearance-related characteristics and the other for personal relationship problems [9].

Transsphenoidal surgery is by far the first line of management; if the surgery is unsuccessful, patients might require adjuvant medical therapy with somatostatin receptor ligands (SRLs), radiotherapy, or both. However, many patients may need primary medical treatment with SRLs if surgery is not feasible [1]. The currently accepted remission criteria of acromegaly are mean 24-hour GH levels < 2.5 μg/l (ng/ml) (5 mU/l) or GH nadir after oral glucose tolerance test (OGTT) < 1 μg/l (ng/ml) (2 mU/l) in the presence of age, gender, and assay-specific normal IGF-I levels [5,10-13]. Random GH >1 μg/l (ng/ml) and GH nadir after OGTT 0.4 μg/l (ng/ml), as well as increased IGF-1, identify active disease biochemically [10]. While most of the recommended biochemical targets aim to improve morbidity and mortality, no cut-off targets aim to enhance life quality; thus, this study aims to investigate the relationship between various hormonal biochemical markers and clinical complaints or findings in patients with acromegaly receiving various treatment modalities.

## Materials and methods

Design and participants

A cross-sectional observational study was done for 56 patients with acromegaly who attended Faiha Specialized Diabetes Endocrine and Metabolism (FDEMC), the tertiary endocrine center in Basrah-Southern Iraq, from September 2020 to September 2021, for biochemical investigations and pituitary imaging, ensuring that no transsphenoidal surgery or radiosurgery was done in the last three months with a response rate of 91.8% (five patients did not consent out of 61). We excluded any pregnant woman and any woman on oral contraceptives in the last three months. Ethical consent was taken in verbal and written form from every participant according to the recommendations of the ethical committee of the College of Medicine, the University of Basrah, with IRB approval number: 2324, on 20/8/2020

Clinical evaluation

Full history was taken by direct interview for age at diagnosis, age at presentation, clinical complaints, and comorbidities, including a history of diabetes mellitus, hypertension, and atherosclerotic cardiovascular diseases, as well as the presence of previous endocrinological disorders. The previous and current treatment modalities were also noted, including any one or combination of TSS, TCS, radiosurgery in the form of Gamma Knife, or medical therapy in the form of SRLs or dopamine agonists.

A collection of clinical complaints related to acromegaly (fatigue, headache, excessive sweating, joint pain, backache, soft tissue swelling, numbness, snoring, and visual problems) were assessed on a four-point Likert scale. The degree of agreement with the items (strongly disagree, disagree, agree, strongly agree) are applied to the scale. These clinical complaints are taken from the SAGIT score [7], AcroQoL [9], and ACRODAT [8].

In addition, complete anthropometric data were obtained regarding body mass index (BMI) by measuring the height, weight, and vital signs, including diastolic and systolic blood pressures.

Laboratory tests

After an overnight fast (eight hours), morning 10 milliliters of venous blood was obtained from all patients, put in tubes with a clot activator, and centrifuged immediately.

For an assessment of biochemical control, dynamic GH testing was done using the electrochemiluminescence technique (Roche cobas®-e411, Germany immunoassay platform) in the form of:

1. Five-point GH curve: where five samples of two milliliters of venous blood at 9:00 am, 10:00 am, 11:00 am, 12:00 pm, and 1:00 pm were taken from 45 patients out of a total of 56 and sent for GH, and the findings to be correlated were nadir, peak and mean GH (from measurements of the five GH readings).

2. For patients on no medical therapy in the last three months, a standard 75 mg OGTT was undertaken the following day. Five samples of two milliliters of venous blood were sent for GH 30 minutes apart (one basal GH and four after oral glucose suppression). The findings to be correlated were nadir, peak, and mean GH of the last four readings.

The GH reference range was > 0.7-50 ng/ml with specified intraassay and interassay precision of ≤6% CV and ≤7% CV, respectively.

Statistical analyses

IBM SPSS Statistics for Windows (Version 26.0. Armonk, NY: IBM Corp.) was used to conduct the statistical analysis. Continuous data were presented as the mean and standard deviation (M ± SD) or mean and standard error of the mean (M ± SE) when appropriate, whereas categorical data were presented as number (N) and percentage (%). To study the difference between the studied symptoms scales and the means of five-point GH curve (mean, peak, and nadir) and GH under OGTT (mean, peak, and nadir), one-way analysis of variance (ANOVA), or Kruskal-Wallis one-way analysis was used when appropriate. While an independent-sample t-test was used in the study, the difference between the biochemical control as defined by 9 am random GH, mean five-point GH curve, and nadir GH under OGTT with the study mean symptoms scales. Statistical significance was defined as a P-value of less than 0.05.

## Results

A total of 56 patients with acromegaly were included in the study; of them, 32 (57.1%) are men. The mean age was 47.8 ± 11.3 years while the mean BMI was 30.7± 5.5 Kg/m². In addition, other data regarding comorbidities, modality of treatments, and all patients' respective characteristics are summarized in Table 1.

**Table 1:** Sociodemographic data and the respective characteristics of patients with acromegaly, comorbidities, and modality of treatments (N = 56) N: number; SD: standard deviation; BMI: body mass index; SBP: systolic blood pressure; DBP: diastolic blood pressure; T2DM: type 2 diabetes mellitus; IHD: ischemic heart disease; SRLs: somatostatin receptor ligands; TSS: trans-sphenoidal surgery; TCS: trans-cranial surgery

Variable	N (%)	Mean ± SD
Sociodemographic data		
Gender (men)	32 (57.1)	
Age (years)	56	47.8±11.3
Age at diagnosis (years)	56	42.4±10.8
BMI (kg/m^2^)	56	30.7±5.5
Comorbidities		
Hypertension	34 (60.7)	
SBP (mmHg)	56	138.8±19.3
DBP (mmHg)	56	83.6±12.6
T2 DM	36 (64.3)	
IHD	6 (10.7)	
Treatment modalities		
Current SRLs	21 (37.5)	
Previous SRLs	29 (51.7)	
One TSS	23 (41)	
Two TSS	2 (3.5)	
TCS	1 (1.78)	
TSS and TSC	1 (1.78)	
Radiosurgery	7 (12.5)	

As described in Table 2, there was no significant difference in biochemical markers in the form of a five-point GH curve (mean, peak and nadir) between different groups of clinical severity with a P-value ≥ 0.05.

**Table 2:** Correlation between clinical complaints and five-point GH curve as a biochemical marker in patients with acromegaly on different modalities of treatment N: number; GH: growth hormone; M ± SE: mean ± standard error of the mean

Symptoms	Score	N	five-points GH curve M ± SE* (ng/dl)			
			Mean	Peak	Nadir	
Fatigue	1	9	5.69 ± 3.32	7.19 ± 4.07	4.82 ± 2.72	
	2	12	6.80 ± 3.08	8.02 ± 3.23	5.87 ± 2.89	
	3	21	5.50 ± 1.89	6.61 ± 2.18	4.65 ± 1.60	
	4	3	9.71 ± 8.28	13.61 ± 11.92	7.63 ± 6.41	
	P-value		0.9	0.9	0.9	
Headache	1	13	7.65 ± 3.05	9.28 ± 3.71	6.48 ± 2.53	
	2	7	3.32 ± 0.80	4.59 ± 1.04	2.55 ±0.70	
	3	19	6.90 ± 2.62	8.25 ±3.01	5.93 ± 2.31	
	4	6	3.93 ± 1.39	5.15 ±1.98	3.25 ± 1.15	
	P-value		0.9	0.9	0.8	
Sweating	1	14	8.17 ± 3.06	9.69 ± 3.55	6.95 ± 2.58	
	2	11	5.90 ± 3.22	6.87 ± 3.30	5.27 ± 3.03	
	3	12	4.69 ± 2.03	6.21 ± 2.93	3.73 ± 1.58	
	4	8	5.23 ± 3.08	6.84 ± 3.86	4.29 ± 2.55	
	P-value		0.8	0.8	0.8	
Arthralgia	1	11	3.65 ± 1.19	4.53 ±1.35	3.09 ± 1.04	
	2	3	2.42 ± 1.10	2.68 ± 1.10	2.21 ± 1.10	
	3	16	8.40 ± 2.91	10.26 ± 3.42	6.98 ± 2.45	
	4	15	6.37 ± 2.78	7.90 ± 3.27	5.48 ± 2.48	
	P-value		0.7	0.6	0.8	
Backache	1	13	7.97 ± 3.40	9.55 ± 3.80	6.87 ± 3.05	
	2	4	3.45 ± 0.71	4.32 ± 0.70	2.99 ± 0.71	
	3	22	2.96 ± 0.66	3.69 ± 0.80	2.50 ± 0.57	
	4	6	15.79 ± 6.17	19.67 ± 7.52	13.03 ± 5.16	
	P-value		0.2	0.1	0.2	
Soft tissue swelling	1	12	3.62 ± 1.11	4.58 ± 1.25	3.04 ± 0.97	
	2	15	4.54 ± 2.01	5.69 ± 2.49	3.76 ± 1.66	
	3	14	4.49 ± 1.81	6.04 ± 2.59	3.66 ± 1.41	
	4	4	25.74 ± 7.72	28.91 ± 8.22	22.56 ± 7.08	
	P-value		0.059	0.07	0.054	
Numbness	1	17	5.94 ± 2.17	7.64 ± 2.66	4.86 ± 1.79	
	2	5	3.08 ± 1.79	3.98 ± 2.58	2.60 ± 1.44	
	3	15	7.44 ± 2.80	9.03 ± 3.28	6.43 ± 2.49	
	4	8	6.17 ± 4.14	6.90 ± 4.51	5.27 ± 3.56	
	P-value		0.5	0.3	0.6	
Snoring	1	6	6.64 ± 5.09	8.57 ± 6.19	5.50 ± 4.20	
	2	8	7.83 ± 3.09	9.78 ± 3.82	6.48 ± 2.60	
	3	19	4.97 ± 1.89	5.94 ± 1.98	4.33 ± 1.77	
	4	12	6.71 ± 3.29	8.16 ± 4.03	5.60 ± 2.73	
	P-value		0.2	0.3	0.3	
Visual disturbances	1	14	7.20 ± 2.67	9.20 ± 3.47	5.93 ± 2.17	
	2	8	3.92 ± 1.12	5.57 ± 1.57	3.16 ± 0.89	
	3	19	6.13 ± 2.47	6.89 ± 2.62	5.36 ± 2.24	
	4	4	7.17 ± 6.43	9.05 ± 8.08	6.06 ± 5.30	
	P-value		0.5	0.4	0.7	

There was no significant difference in the GH under 75 gm OGTT (mean, peak, and nadir) between different groups of clinical severity apart from backache with a highly significant difference (P-value <0.01, <0.01, and <0.01), respectively, as shown in Table 3.

**Table 3:** Correlation between clinical complaints and GH under OGTT as a biochemical marker in patients with acromegaly on different modalities of treatment N: number; OGTT: oral glucose tolerance test; GH: growth hormone; M ± SE: mean ± standard error of the mean

Symptoms	Scale	N	GH under OGTT M ± SE* (ng/ml)			
			Mean		Peak	Nadir
Fatigue	1	5	1.85 ± 0.63		2.21 ± 0.77	1.58 ± 0.54
	2	5	6.09 ± 2.07		7.81 ± 2.87	4.13 ±1.29
	3	16	6.11 ± 1.86		6.77 ± 1.95	5.11 ± 1.65
	4	5	5.86 ± 3.61		6.72 ± 4.04	4.61 ± 3.00
	P-value		0.3		0.4	0.4
Headache	1	9	8.18 ± 3.24		9.23 ± 3.49	6.88 ± 2.85
	2	3	3.86 ± 0.90		4.57 ± 1.23	2.47 ± 0.43
	3	15	4.14 ± 1.22		4.88 ± 1.42	3.22 ± 0.96
	4	4	4.90 ± 2.62		5.52 ± 2.64	3.94 ± 2.19
	P-value		0.7		0.8	0.8
Sweating	1	9	6.14 ± 2.95	6.94 ± 3.16		5.18 ± 2.62
	2	8	4.56 ± 1.27	5.16 ± 1.32		3.68 ± 1.04
	3	8	6.21 ± 2.27	7.38 ± 2.63		4.55 ± 1.86
	4	6	4.23 ± 2.74	4.88 ± 3.01		3.49 ± 2.30
	P-value		0.5		0.6	0.6
Arthralgia	1	7	3.48 ± 1.31	4.45 ± 1.81		2.61 ± 0.81
	2	3	2.23 ± 1.09	2.53 ± 1.28		1.98 ± 0.91
	3	10	8.21 ± 2.72	9.24 ± 2.89		6.75 ± 2.44
	4	11	4.88 ± 1.85	5.53 ± 2.03		3.79 ± 1.53
	P-value		0.2		0.2	0.2
Backache	1	6	3.51 ± 1.67	4.41 ± 2.20		2.62 ± 1.09
	2	2	4.30 ± 0.55	4.84 ± 0.69		3.31 ± 0.24
	3	19	3.42 ± 0.76	4.04 ± 0.91		2.65 ± 0.57
	4	4	18.04 ± 4.44	19.77 ± 4.42		15.17 ± 4.39
	P-value		0.01		0.01	0.01
Soft tissue swelling	1	8	3.50 ± 1.13	4.34 ± 1.57		2.70 ± 0.69
	2	10	3.05 ± 0.66	3.64 ± 0.79		2.25 ± 0.51
	3	10	5.11 ± 1.98	5.69 ± 2.18		4.14 ± 1.63
	4	3	19.11 ± 4.92	21.30 ± 4.31		15.95 ± 5.22
	P-value		0.07		0.06	0.07
Numbness	1	9	4.98 ± 1.91	5.84 ± 2.21		3.99 ± 1.51
	2	5	1.97 ± 0.66	2.28 ± 0.76		1.67 ± 0.56
	3	9	6.72 ± 2.14	7.85 ± 2.41		5.30 ± 1.75
	4	8	6.47 ± 3.20	7.18 ± 3.32		5.18 ± 2.93
	P-value		0.3		0.3	0.4
Snoring	1	6	2.33 ± 0.50	2.71 ± 0.59		2.01 ± 0.42
	2	5	8.07 ± 2.87	9.63 ± 3.30		6.04 ± 2.37
	3	11	3.42 ± 0.94	4.21 ± 1.23		2.45 ± 0.61
	4	9	8.33 ± 3.28	9.04 ± 3.47		7.12 ± 2.91
	P-value		0.3		0.3	0.3
Visual disturbances	1	9	5.62 ± 2.00	6.69 ± 2.33		4.53 ± 1.59
	2	4	5.17 ± 2.51	5.77 ± 2.52		4.26 ± 2.07
	3	14	5.44 ± 1.92	6.23 ± 2.06		4.23 ± 1.71
	4	4	4.86 ± 4.29	5.37 ± 4.72		4.09 ± 3.58
	P-value		0.5		0.5	0.4

Furthermore, this study investigates the correlation between the mean of symptoms' scale with different cut-offs for biochemical control.

**Figure 1:**
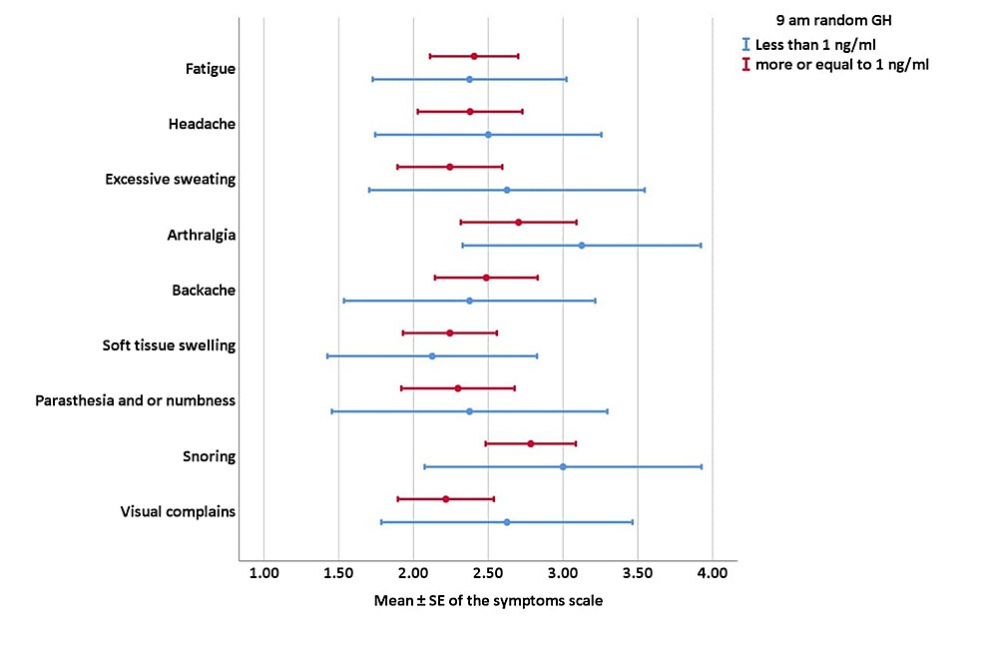
Correlations between the 9 am random GH target and symptoms’ scale GH: growth hormone; SE: standard error of the mean

As shown in Figure 1, there was no significant difference in the mean of the symptoms scale of all symptoms between patients with controlled and uncontrolled 9 am random GH (P-value ≥ 0.05).

**Figure 2:**
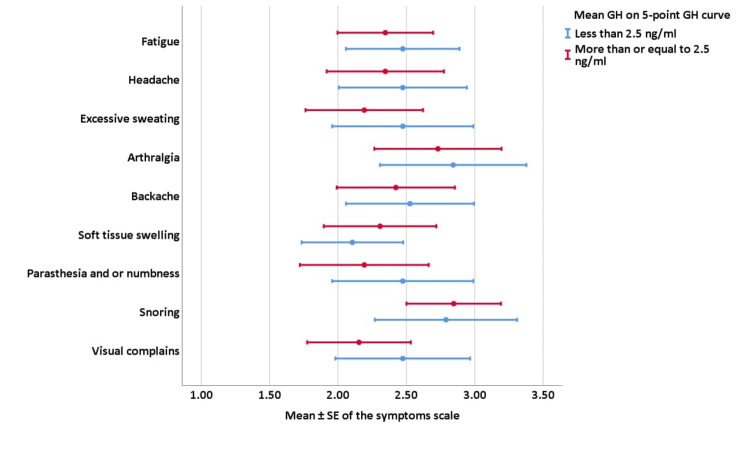
Correlations of target GH mean on five-points GH curve with symptoms’ scale. GH: Growth hormone; SE: Standard error of the mean

As shown in Figure 2, the same finding was seen after assessment of mean GH on five-point GH curve cut-off, where our statistics did not find a significant difference in the mean of the symptoms scale of all symptoms between patients with five-point GH mean less than or equal to 2.5 ng/ml and those with more than 2.5 ng/ml (P-value ≥ 0.05).

**Figure 3:**
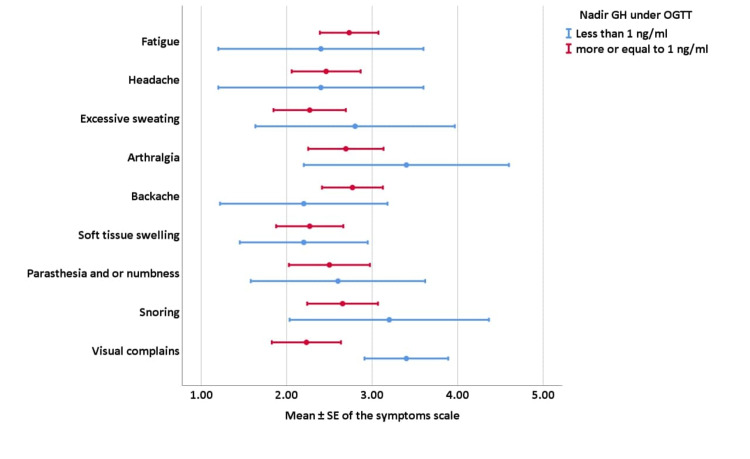
Correlations of target GH nadir on GH under OGTT with the symptoms’ scale GH: growth hormone; OGTT: oral glucose tolerance test; SE: standard error of the mean

Likewise, the picture was similar in the patients’ group with nadir GH under OGTT below and above 1 ng/ml, as shown in Figure 3, which describes the correlation between the mean symptom scale of all symptoms and the mean nadir GH under OGTT (P-value ≥ 0.005).

## Discussion

This study investigates the most crucial aspect of acromegaly patients’ lives: their symptomatology and quality of life. The present study shows a mean age of presentation and diagnosis of 42.4 ± 10.8, which was consistent with the previous local and international data of 46.4 ± 10.8 and 44, respectively [14-15].

There was no correlation between any of the studied clinical complaints with biochemical markers apart from backache, consistent with multiple previous studies [16-17].

In 2020, Geer et al. found, in their prospective study on 105 patients on SRL therapy, a persistence of their clinical complaints while on treatment [16]. For example, 80% of the studied patients had four symptoms with constant experience of fatigue in 77%, snoring in 74%, acro-fog in 71%, vision problems in 67%, joint pain in 66%, carpal tunnel syndrome in 60%, excessive sweating in 55%, soft tissue swelling in 48%, and headache in 41%.

In their prospective study on 58 acromegaly patients treated with SRL (octreotide long-acting release (LAR)), Chin et al. in Korea (2015) found no significant correlation between biochemical markers and HrQoL assessed by their validated version of AcroQoL apart from psychological appearance [17].

Furthermore, to assess the severity of the clinical symptoms in biochemically controlled status, the current study showed no correlation between different clinical complaint scales with biochemical control targets (P-value > 0.05). This was in concordance with Geer et al. in 2020, where a constant experience of acro-fog in 89%, fatigue in 82%, snoring in 68%, carpal tunnel syndrome in 67%, joint pain in 64%, and vision problems in 61% was noticed after control [16]. For patients on SRLs (lanreotide auto gel), a study in Taiwan (2006) also found the same results with no significant difference between the controlled and uncontrolled groups [18]. Even though the previous two studies performed on patients receiving SRLs, the non-significant difference between controlled and uncontrolled groups and persistence of symptoms after biochemical control emphasize the generalization of the idea of clinical and biochemical mismatch.

In a Belgian study done by T’Sjoen et al. (2007), a cross-sectional evaluation of 291 patients to evaluate their AcroBel questionnaire (the Belgian version of AcroQol) and signs and symptoms score (SSS), which is a disease-specific tool that assesses five common complaints in acromegaly: headache, arthralgia, soft tissue swelling, excessive sweating and fatigue, they found no correlation between biochemical markers of active disease and HrQoL [19]. Regarding SSS, only soft tissue swelling significantly correlates with disease activity defined by GH and IGF-1 levels. The same finding was found previously by Rowles et al. in 2005, where the SSS shows no relationship with GH and IGF-1 values in their sample of 80 patients [20].

On the other hand, some studies confirm an improvement in clinical complaints and HrQoL after biochemical control. For example, in 2020, a Chinese study concluded that there is improvement in patients’ symptoms and HrQoL after patients' control [21]. However, despite the statistically significant reduction in their symptom frequency after biochemical control, the persistence of these symptoms was demonstrated in a significant proportion.

Moreover, German and Japanese studies in 2007 [22] and 2016 [23] found the same results with improvement in clinical signs and symptoms after starting pegvisomant therapy; however, the previous three studies were designed to assess improvement in symptoms after treatment rather than persistence or comparison between patients with controlled and uncontrolled disease as with the present study, which assesses, using a cross-sectional design, the real-life correlation between different clinical and biochemical parameters at a point in time.

In contrast, other studies show a different view; in Shanghai, China, Gu et al. (2020) concluded that improvement in HrQoL was achieved in acromegaly patients treated by transsphenoidal surgery independent of biochemical control [24]. Negger et al. (2008) found in their prospective, double-blind placebo-controlled study on 20 patients on SRLs therapy that there is an improvement in their HrQoL after adding pegvisomant to SRLs with no significant reduction even in patients with controlled baseline IGF-1 level [25]. These findings support the idea that HrQoL does not correlate with biochemical control, and other factors like the patient’s selection, personal characteristics, and type of treatment can have the dominant hand in the game.

Limitations

First, IGF-1 was not used as a biochemical marker of disease control because of the lack of test standardization and the shortage of tests in the last months of the study. Second, the number of patients was influenced by the availability of drugs and investigations especially in the era of emerging COVID and curfew, which affected the number, and finally, the subjective nature of the clinical data collection may affect our results, as some patients may find simple headaches or other symptomatology bothersome for them; however, using a Likert scale rather than a yes or no questionnaire may abolish this problem.

## Conclusions

There was no correlation between different biochemical markers in the form of a five-point GH curve mean, peak and nadir; GH under OGTT suppression mean, peak, and nadir, and different patients’ complaints apart from backache where a significant association was found only in the GH suppression under OGTT parameters.

No significant differences were found in this study regarding the severity of clinical complaints between biochemically controlled and uncontrolled groups of patients when using different cut-off values (less than 1 ng/ml random 9 am GH, less than 2.5 ng/ml mean five-point GH curve, and 1 ng/ml nadir GH under OGTT suppression) as biochemical control markers. While different biochemical control targets were developed previously to decrease morbidity and mortality in patients with acromegaly, it appears in this study that the severity of the clinical symptoms does not correlate with biochemical control so that thorough evaluation and treatment of clinical complaints and HrQol beside biochemical control is an essential part of the management plan in patients with acromegaly.
